# Tissue oxygenation: how to measure, how much to target

**DOI:** 10.1186/s40635-023-00551-1

**Published:** 2023-09-23

**Authors:** Matthias P. Hilty, Christian Jung

**Affiliations:** 1 Institute of Intensive Care Medicine, University Hospital of Zurich, Rämistrasse 100, 8091 Zurich, Switzerland; 2 Medical Faculty, Department of Cardiology, Pulmonology and Vascular Medicine, Heinrich-Heine-University Duesseldorf, Moorenstraße 5, 40225 Duesseldorf, Germany

**Keywords:** Tissue oxygenation, Tissue perfusion, Microcirculation, Hemodynamic management, Fluid management, Circulatory shock, Resuscitation, Vasopressors

Circulatory shock, defined as a life-threatening, generalized form of acute circulatory failure in which the circulation is unable to deliver sufficient oxygen to meet the demands of the tissues, remains a major contributor to morbidity and mortality in critically ill patients. Hemodynamic management traditionally focuses on increasing global oxygen supply, most commonly through optimization of stroke volume and cardiac output. While early guidelines have mostly recommended to maximally utilize the oxygen-carrying capacity of the blood, recent studies have put oxygenation targets in the spotlight in the management of critically ill patients in circulatory shock. Several aspects underline the importance of thoughtful and individualized (re-)consideration of oxygenation targets [1]. First, in high hemoglobin oxygen saturation ranges (e.g., > 90–92%), an increase of inspired oxygen fraction—due to the nature of the hemoglobin oxygen binding curve—primarily promotes higher arterial oxygen partial pressure and only marginally contributes to the oxygen-carrying capacity of the blood and global delivery of oxygen. It is, on the other hand, increasingly recognized that such hyperoxia conveys harmful side effects associated with reactive oxygen species and changes in microcirculatory function. Second, adaptation to (mild) hypoxia, when correctly balanced to avoid the threshold to precipitate cellular dysfunction and organ failure, may take place in the context and timeframe of critical illness as recently demonstrated in COVID-19 patients [2], and may contribute to a positive risk–benefit ratio.

The discussion of oxygenation targets inevitably raises the question of considering global versus tissue-centered resuscitation goals. While current practice mostly targets global delivery of oxygen, emerging techniques to quantify tissue perfusion and oxygenation place the direct targeting of determinants of tissue oxygenation within reach of the clinician. Tissue perfusion, which essentially describes the movement of hemoglobin carriers through the capillaries, or, in other words, the perfusion of the tissue with red blood cells [3], depends on the diffusion and convection capacity of oxygen in the tissue. These, in turn, are dependent on the individually regulated capillary density and capillary hematocrit, and red blood cell velocity. In addition, tissue oxygenation depends on the oxygenation of the individual oxygen carriers, and the capacity of the tissue to extract oxygen. In this context, oxygen delivery depends on balancing resuscitation measures and disease conditions with effects such as hemodilution, tissue edema, arteriolar constriction and venular tamponade, and oxygen extraction additionally depends on the presence of microcirculatory shunts and mitochondrial dysfunction [4]. Such a tissue-centric approach provides a novel insight into the physiology and pathophysiology of tissue oxygenation, and also on the effects of hypoxia and hyperoxia on these functional components of the microcirculation [5].

The thematic collection “Tissue oxygenation: how to measure, how much to target” invites authors to focus on diagnostic and therapeutic aspects of a direct view on the determinants of tissue oxygenation. The aim is to not only contribute to a tissue-centric perception of the concept of oxygen delivery and extraction, but also to set the stage for taking the next step to improve the management in critically ill patients suffering from circulatory shock.
